# Beneath the surface: a survey evaluating ASF preparedness across the Caribbean and Latin America

**DOI:** 10.1186/s13567-026-01886-9

**Published:** 2026-11-13

**Authors:** Rachael Schulte, Michelle L. Schultze, Guillermo Arcega Castillo, Luis Pablo Hervé-Claude, Emilio A. León, Andres M. Perez

**Affiliations:** 1 Center for Animal Health and Food Safety, College of Veterinary Medicine, University of Minnesota, St. Paul, MN 55108 USA; 2 Animal Disease Control Branch, Hawaii Department of Agriculture and Biosecurity, Honolulu, HI 96814 USA; 3 Department of Biomedical Sciences, Ross University School of Veterinary Medicine, Basseterre, Saint Kitts and Nevis; 4 Department of Clinical Sciences, Lewyt College of Veterinary Medicine, Long Island University, 720 Northern Blvd, Brookville, NY 11548 USA

**Keywords:** Veterinary services, emergency response and planning, swine diseases, transboundary diseases, West Indies, mixed methods, swine, disease preparedness

## Abstract

African swine fever (ASF) threatens pig production across the Americas, yet empirical data on preparedness capacity remain sparse. To inform regional planning and capacity building, we conducted a comparative assessment of ASF preparedness across countries and territories (hereafter “territories”) in the Caribbean basin. The final sample size was 18 territories, 41% of the 44 territories identified as the target population in and around the Caribbean basin. Via semi-structured interviews, respondents completed a 0–10 agreement-scale questionnaire investigating ASF preparedness. Quantitative analysis was used to construct a territory-by-statement matrix and Spearman correlation matrix. Qualitative data were analyzed inductively. A wide range of preparedness scores were found, with the highest preparedness score observed for the presence of legislative authority to prevent and respond to ASF incursion, and the lowest score observed for the characterization of the feral swine population. Strong positive correlations were identified between contingency planning and operational readiness; wildlife and risk-based surveillance; and prevention regulations, ASF governance, and producer communication. Additionally, a thematic analysis was performed to identify specific gaps in preparedness; gaps were found in communication and coordination, pig production system characterization, prevention and control, resources, surveillance, and traceability. Site visits were performed to validate the questionnaires. Our study shows gaps in preparedness throughout the region, most noticeably in the Caribbean island territories, that can be used to inform future capacity building efforts within territories and the region. Capacity building efforts should take into consideration the needs of the requesting territory as well as the level of impact the proposed intervention would have.

**Supplementary Information:**

The online version contains supplementary material available at https://doi.org/10.1186/s13567-026-01886-9.

## Introduction

African swine fever  (ASF) is a transboundary viral disease of pigs that imposes substantial socio‑economic losses worldwide [1]. Its causative agent, African swine fever virus  (ASFV), produces an acute haemorrhagic syndrome that is usually lethal in domestic pigs and in most wild suids [2, 3]. Since the disease was first documented in 1921 [4], it has remained an enduring threat to the global pig industry [1].

Although transmission dynamics differ among countries and production systems, ASFV can spread rapidly through direct contact with infected animals, contaminated fomites or pork products, and via Ornithodoros soft tick bites [5–10]. Active outbreaks now occur in Africa, Europe, Asia, Oceania and the Caribbean, underscoring ASF’s status as one of the principal hazards facing livestock production [11–18]. On Hispaniola, the virus re‑emerged in July 2021 when cases were confirmed in the Dominican Republic, followed by Haiti two months later [11, 19]. This resurgence came nearly four decades after the island had been declared free of ASF [20–22].

Regional international organizations such as the Caribbean Animal Health Network (CaribVET), the Caribbean Agricultural Health and Food Safety Agency (CAHFSA), the French Agricultural Research Centre for International Development (CIRAD), the Organismo Internacional Regional de Sanidad Agropecuaria (OIRSA), and the Inter-American Institute for Cooperation on Agriculture (IICA) provide support to member countries facing livestock disease challenges. CaribVET supports regional surveillance efforts by providing capacity building around surveillance, early warning, and response to livestock diseases and diagnostic capacity [23, 24]. CAHFSA was created by the member states of the Caribbean Community (CARICOM), a regional economic and political union of twenty-one countries in the Caribbean, to support the region in development of a sanitary and phytosanitary (SPS) framework and to provide support to the region and member states in establishing and managing agricultural systems, and in developing a harmonized animal health standard in the region [24–26]. CIRAD is a research organization that works to support agriculture in the Caribbean and Mediterranean regions; it provides research support for animal and human health in addition to other avenues of agricultural research [27]. OIRSA provides technical and financial assistance to member states to promote development of agriculture and food safety; it has provided significant assistance in preventing exotic animal diseases from entering the region, and in eradication of economically significant pests and diseases from the region [28]. IICA focuses on agricultural development, supporting innovation and capacity building in the region in agricultural health and food safety, as well as other areas of agricultural development [29]. These regional international organizations provide significant support to their member territories in the areas of disease detection, prevention, control, and eradication, and protecting animal and human health through surveillance, improved legislation and policy advocacy, and fostering collaboration between member states to improve the overall preparedness of the region.

The Caribbean basin and the nations of Central and South America are linked to the United States by intense tourism, commercial shipping and informal trade in pork products [30–34]. Although valuable qualitative reports and expert consultations are available, comprehensive, territory‑level empirical data on the region’s ability to prevent, detect and control ASF remain limited [30, 31, 35–37].

To contribute to understanding regional capacity to respond to ASF, the present study aims to compare the preparedness capacity of forty‑four Caribbean, Central‑ and South‑American territories. Specifically, the study was aimed to measure the region’s resources, coordination and operational readiness, describe their susceptible‑population profiles and existing ASF programmes, and analyze qualitative feedback to identify common deficiencies in prevention, detection and outbreak management. From these data we generate a composite preparedness score for each territory and use it to compare preparedness patterns across the region. By revealing strengths and weaknesses, this investigation will provide important context to competent national and regional authorities that prioritise surveillance, allocate technical assistance and design targeted capacity‑building initiatives, allowing interventions to be further customized to each territory and region.

To achieve these aims, we conducted structured interviews with veterinary‑service representatives from the participating territories, where respondents were invited to complete a questionnaire assessing resources, coordination and operability of their territory’s ASF preparedness. Respondents were also invited to provide additional comments to provide more context on their territory’s unique situation. Quantitative and qualitative analyses were then performed on the data collected from the interviews. This mixed‑methods design ensures that measurable indicators are complemented by nuanced insights, providing a robust foundation for the objectives and hypotheses outlined above.

## Materials and methods

This study is a follow‑on investigation within a broader research programme that has already produced a qualitative risk‑classification evaluating the likelihood of ASF incursion across forty-four Caribbean, Central‑ and South‑American countries and territories (hereafter “territories”). In that initial publication, our multi‑disciplinary team conducted a systematic, multi‑stage review of scientific papers, government documents, grey literature and international epidemiological databases, assessing the risk of ASF incursion through informal pork imports and legal commercial imports for each territory [36].

The present work builds on those findings by shifting the focus from pathway likelihood to regional preparedness. Territories located in and around the Caribbean basin were defined as the study population and were invited to participate. Forty-four territories were included in the study population: Anguilla, Antigua and Barbuda, Aruba, Bahamas, Barbados, Belize, Bermuda, Bonaire, British Virgin Islands, Cayman Islands, Colombia, Costa Rica, Cuba, Curaçao, Dominica, the Dominican Republic, El Salvador, Grenada, Guadeloupe, Guatemala, Guyana, Haiti, Honduras, Jamaica, Martinique, Mexico, Montserrat, Nicaragua, Panama, Puerto Rico, Saba, Saint Barthelemy, Saint Lucia, Saint Martin, Sint Eustatius, Sint Maarten, St Kitts and Nevis, St. Vincent and the Grenadines, Suriname, Trinidad and Tobago, Turks and Caicos, US Virgin Islands, Venezuela. St Kitts and Nevis, at the request of a collaborator, were interviewed separately. Employing a mixed‑methods design, we carried out semi‑structured interviews with senior veterinary‑service officials from participating territories, supplemented by a Likert‑scale questionnaire and open‑ended narrative comments. This approach generated empirical data on prevention resources, surveillance infrastructure, outbreak‑response capacity and the regulatory environment—dimensions that were previously described only qualitatively—thereby filling a critical evidence gap for regional ASF risk management. These data were also used in a related article that assessed the connectivity of Caribbean countries as a risk factor for ASF incursion [38].

### Questionnaire development

A Likert-scale questionnaire was developed to assess each target territory’s current capacity to prevent, detect, and control ASF. Questions were phrased as statements, and respondents were asked to assign a number between 0 and 10 reflecting agreement with the statement for their territory, with 0 meaning complete disagreement and 10 meaning complete agreement. The statements covered three topics: resources, coordination, and operativity; susceptible population; and pig health and ASF program (mapped as S1-S5; S6-S10; S11-S30, see Additional File 1). There was an additional section with statements pertaining to control efforts that only countries with active ASF outbreaks were asked to complete. Respondents were also invited to provide comments on each statement to further clarify their answers and provide additional context. When a statement did not apply to a territory’s context (e.g. the statement asked about feral pigs, but the territory does not have a feral pig population), respondents could mark it as not applicable (N/A); these entries were retained as missing for analysis and displayed as N/A in tables and figures. Following development of the questionnaire, it was tested in a pilot exercise, with the participation of 8 veterinarians from the target territories. The pilot exercise resulted in refinement of the 0–10 response scale and clarification of the wording of some statements. No weighting was applied to questionnaire statements; all quantitative analyses used the original, unweighted 0–10 agreement scores. Two coordination meetings were organized to standardize the interview methodology among interviewers.

The questionnaires were provided to respondents ahead of time, to give them time to gather information from relevant sectors. Meetings were then conducted via Zoom (territories 1–13) or in-person during site visits (territories 14–18) with an interviewer and a representative from the territory’s Veterinary Services or corresponding government agency; invitations were also extended to representatives of the swine sector, but such representation was possible in only one territory. Interviews were conducted in English or Spanish, lasted approximately 2 to 2.5 h, and data were collected in the same language in Microsoft Excel [39] spreadsheets. Comments were then translated into English for analysis. The survey was open from October 2024 through June 2025. As no identifiable private information about participants or biospecimen samples were collected, this work was declared as non-human research by the University of Minnesota Institutional Review Board (#STUDY00023566). All participants provided consent to participate, and participation could be revoked at any time.

### Sample frame

We targeted a census of eligible jurisdictions. Forty-four countries and territories across North America, Central America, South America, and the Caribbean were invited to participate. The unit of analysis was the territory-level competent veterinary authority, with one consolidated response per territory. Inclusion criteria were: (i) formal designation by the competent authority as an official responsible for animal health (e.g., Chief veterinary officer, extension officer, agricultural officer, or a delegated representative), and (ii) capacity to provide a territory-level response covering ASF preparedness.

### Statistical analysis

We analyzed questionnaire ratings on the original 0–10 agreement scale; items marked not applicable (N/A) were retained as missing for all computations.

**Figure 1:**
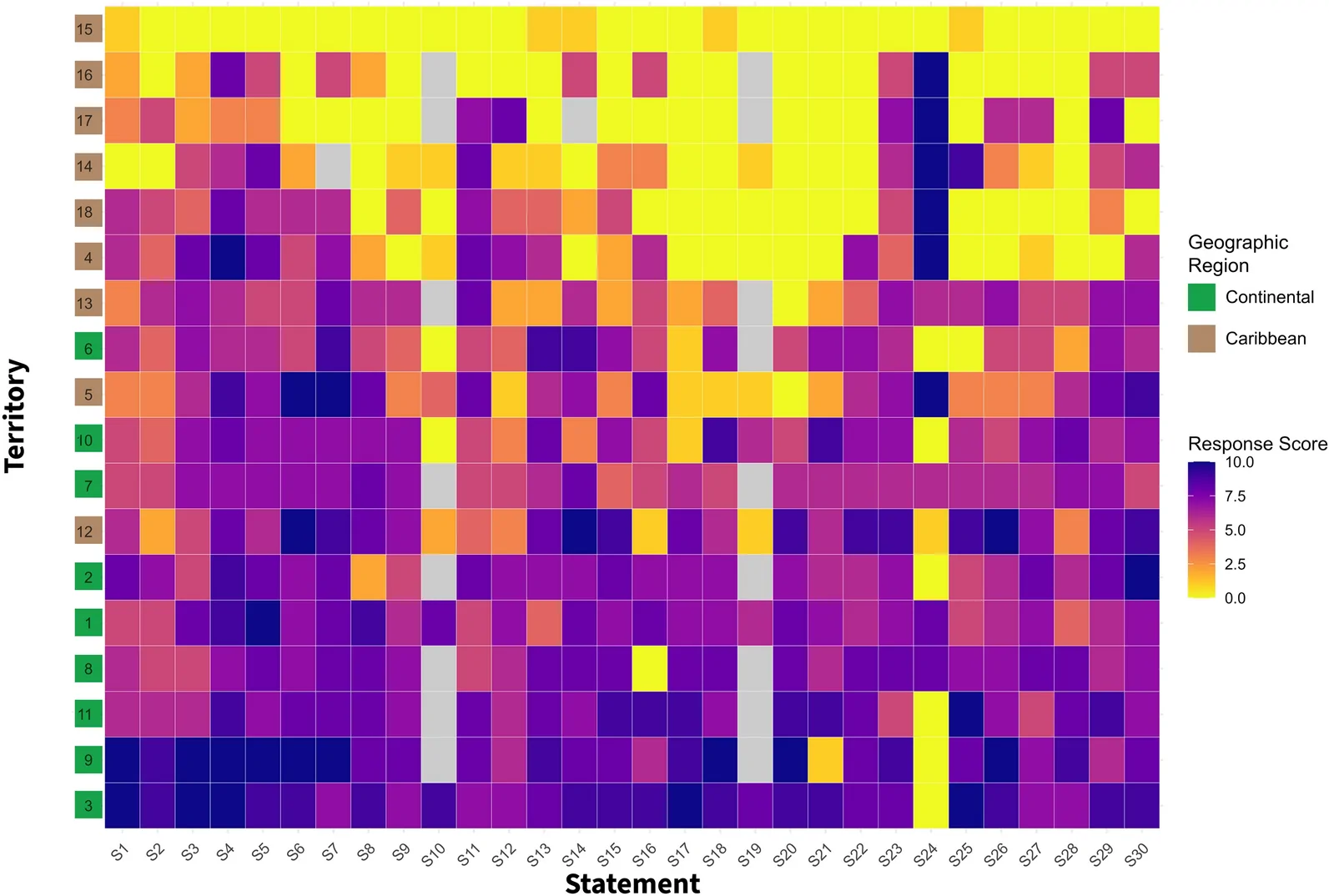
**ASF preparedness responses by territory: heatmap of ASF preparedness ratings on a 0–10 agreement scale by territory (rows) and statements SQ1-SQ30 (columns), with territories ordered by mean score (in descending order).** Colored bars indicate geographic classification (green: Caribbean; brown: Continental America). Low scores (0–3) indicate low agreement/moderate preparedness, moderate scores (4–7) indicate moderate agreement/moderate preparedness, and high scores (8–10) indicate high agreement/high preparedness. Grey cells indicate statements marked as not applicable (N/A) and were treated as missing values in the analysis.

We constructed a territory by statement matrix for the 30 universally administered items (S1–S30). Territories are ordered by their unweighted mean score across non-missing items (descending), and statements are displayed in questionnaire order. Values are plotted on the original 0–10 scale using a heatmap from low (purple, 0–3) through mid (orange, 4–7) to high (yellow, 8–10), with N/A shown in grey (see Figure 1). To quantify concordance among indicators, we computed pairwise Spearman rank correlations (ρ) using pairwise-complete observations, classifying coefficients of 0.80 or higher as highly positive, –0.50 or lower as strongly negative, and values between –0.20 and 0.20 as indicating little to no association.

### Internal consistency

To evaluate internal consistency, we evaluated conceptually linked sets of statements (e.g., funding for surveillance and laboratory capacity) using two complementary screening tests in parallel: (i) categorical alignment after partitioning ratings into Low (0–3), Moderate (4–7), and High (8–10); and (ii) within-set dispersion, using the standard deviation ≥ 2 as potentially inconsistent. Three sets of linked statements were identified (see Additional File 2). Items flagged by either method as inconsistent were reviewed alongside narrative comments to determine whether contextual factors plausibly explained inconsistencies. All procedures used only non-missing values.

All data analysis and visualization were performed in R version 4.4.1 using the corrplot package [40, 41] with standard plotting libraries and SAS 9.4 [42]; primary data capture occurred in Microsoft Excel [39].

### Thematic analysis

A thematic analysis was performed using Nvivo software [43]. The goal of this analysis was to identify common gaps in prevention, detection, and outbreak response capabilities of responding territories. An inductive stepwise analysis was performed on provided comments following the methods presented by Braun and Clarke [44]. First, responses were divided among three reviewers such that all responses were reviewed by two team members. Reviewers independently reviewed all survey responses to become familiar with the data before generating initial codes; patterns were noted for potential themes. Once the initial codes were generated, responses were reviewed for consistency with assigned codes, and codes were aggregated into overarching themes. Themes were critically evaluated in the context of survey responses to ensure relevance. Finally, independently generated codes and themes were compiled and compared for consistency. For responses that were coded inconsistently, a structured discussion was used to achieve consistency.

### Proposed capacity building mitigation strategies

In addition to responding to questionnaire statements and providing contextual responses, territories were invited to provide mitigation strategies that would be of greatest benefit to their territory; the following categories were provided for respondents to list strategies within: (1) training, (2) equipment, supplies, and logistical support, and (3) infrastructure. The proposed mitigation strategies were compiled in Microsoft Excel [39] and organized by the provided categories. The strategies listed by respondents were then categorized into groups (i.e. mitigation strategies focusing on training, simulation exercises, and outbreak drills were categorized into one group), and the percentage of territories listing each type of strategy was calculated.

### Site visit observations

Site visits were conducted between May 2025 and June 2025 to five territories: 14, 15, 16, 17, and 18. Territories were chosen based on a representative from the veterinary services availability, willingness to host a reviewer, and schedule availability. Three authors split travel over two separate trips with multiple site visits each trip to minimize the amount of travel required per site visit; on average, each site visit lasted one day. Reviewers traveled from St. Kitts and Minneapolis, and met with one to three government representatives at each site visit; visits to farms, landfills, ports of entry, veterinary services offices and other sites were included. These visits involved gathering first-hand experience from one of three reviewers in these territories to assess the validity of the questionnaire responses. Reviewers were shown around each territory by a representative from their territory’s veterinary services. During these visits observational notes and discussion with the visit participant were taken on governance structure, swine production and livestock management, regulatory environment, disease management and surveillance, environmental components, regional communication gaps, and recommended actions. This information was used to validate the questionnaire, by comparing the site visit observations with the answers to related statements on the questionnaires.

## Results

Of the 44 eligible jurisdictions in the study population, 18 territories agreed to participate, resulting in a 41% response rate. The sample population included 9 Caribbean islands (territories 4, 5, 12–18) and 9 countries bordering the Caribbean in Central and South America (hereafter referred to as the continental territories; territories 1–3, 6–11).

### Statistical analysis

Perceived mean preparedness on the 0–10 scale (unweighted across items; N/A excluded from means) ranged from 8.33 to 0.17. The highest values occurred in territory 3 (8.33), territory 9 (8.04), territory 11 (7.32), and territory 8 (6.89). The lowest values were observed in territory 15 (0.17), territory 16 (2.11), territory 17 (2.52), and territory 14 (2.76). The ordering and intensity patterns are shown in Figure 1, while the item-level responses for each territory are provided in Additional File 3.

Across the 30 statements, the highest mean score was observed for S4 in the resources, coordination, and operativity domain (“There is a legislative framework that grants sufficient authority to the veterinary authority to enact the required measures and provide adequate support”; mean score: 7.39), with S5 in the same domain (“The private pig industry is positive and receptive to the ASF control measures enacted by the Veterinary Authority”; mean score: 6.67), S7 in the susceptible population domain (“Commercial pig producers are registered, and their location and pig stocks are known”; mean score: 6.88), and S23 in the swine health and ASF program domain ((“Farmers and veterinarians (private and public) are well-trained to detect and respond to ASF and commonly report suspicious clinical signs/mortality to the Veterinary Authority for investigation”; mean score: 6.28). The lowest means were recorded for S10 in the susceptible population domain (“The population of wild boars and feral pigs in the country has been studied and characterized”; mean score: 2.50) and S19 in the swine health and ASF program domain (“Surveillance activities regularly include the country's wild boar and feral pig populations”; mean score: 2.56), with additional low values for S17 in the swine health and ASF program domain (“A contingency plan exists to respond to an ASF emergency, which has been tested in at least one simulation exercise”; mean score: 3.83) and S21 in the same domain (“High-risk populations are identified, and specific, risk-based surveillance is periodically carried out in these populations”; mean score: 3.89). For territories that stated there were no wild suids present, their responses to S10 and S19 were recorded as N/A.

Building on these item level means, we examined pairwise associations using Spearman’s rank correlation (ρ), a nonparametric measure of monotonic association ranging from − 1 to 1 (see Additional File 4). Several strong positive associations were identified among preparedness indicators. Contingency planning and operational readiness were highly correlated: contingency plan testing (S17) correlated very highly with laboratory capacity, field logistics for biosecurity and disinfection, and passive surveillance (S20, S26, and S18) (ρ = 0.93, 0.88, and 0.83, respectively). National ASF plan/governance (S15) aligned with laboratory capacity and contingency plan testing (S20 and S17) (ρ = 0.90 and 0.87, respectively). Another pattern involved wildlife and targeted surveillance: routine wild suid surveillance (S19) correlated very strongly with surveillance in high-risk populations, swill/feed regulation, and with high levels of biosecurity practices reported (S21, S27 and S28) (ρ = 0.91, 0.88, and 0.85, respectively). Disease-prevention regulations (S13) were also closely related to both existing communication plans to alert producers about ASF and national ASF plan/governance (S22 and S15) (ρ = 0.89 and 0.83, respectively), indicating coherence among ASF prevention, communication, and governance measures. The most negative relationships centered on absence of differential pig diseases (S24), which was inversely associated with several programmatic capacities, including passive surveillance, disease-prevention regulations, risk-based surveillance, laboratory capacity, contingency plan testing, backyard pig population studied, and national ASF plan/governance (S18, S13, S21, S20, S17, S9, and S15) (ρ =  − 0.78, − 0.65, − 0.65, − 0.63, − 0.60, − 0.58, and − 0.58, respectively). Several item pairs showed little to no association, suggesting independence in practice. For example, formal slaughter with inspection (S11) had multiple near-zero correlations with other program indicators.

### Internal consistency

Using both methods, those territories that were inconsistent in each pair of statements were identified (see Table 1). There were a total of nine territories that had one or more inconsistencies in their responses. Discussion between lead researchers and collaborators on this project attempted to identify reasons for these inconsistencies based on their expertise and knowledge of the territory; when rationale was identified, it was included (see Table 1). For S2 (availability of funds) and S18 (passive surveillance), there were six territories with inconsistent responses. Explanations for inconsistencies were identified for two of the territories (see Table 1). For S2 (availability of funds) and S20 (laboratory capacity), inconsistencies were identified for seven territories. Two of these territories had no laboratory capacity and therefore responded low to S20, but had funds for other measures in S2. Another territory had previous external funding to build up their laboratory capabilities but other funding measures were insufficient. For S1 (sufficient staffing), S18 (passive surveillance), S2 (availability of funds), and S26 (disinfection and biosecurity supplies and protocols), there were three territories with inconsistencies in each pairing. For the last pair of statements, S10 (wild pigs profiled) and S19 (wildlife surveillance), inconsistency was identified in one territory. Two territories (18 and 4) accounted for four different inconsistencies each, but only one territory’s responses provided explanations for each inconsistency.

**Table 1:** **Territories with inconsistencies**

Group 1	Statement pairs		Reason for inconsistency
*Territory*	*S2*	*S18*	
Territory 18	5/Mod	0/Low	No reason identified
Territory 10	4/Mod	9/High	No reason identified
Territory 12	2/Low	6/Mod	No reason identified
Territory 8	5/Mod	8/High	No reason identified
Territory 4	4/Mod	0/Low	No surveillance. Also S18 is linked to collection and analysis of samples, and there is no lab
Territory 17	5/Mod	0/Low	No surveillance. Also S18 is linked to collection and analysis of samples, and there is no lab
*Territory*	*S2*	*S20*	
Territory 18	5/Mod	0/Low	No reason identified
Territory 1	5/Mod	8/High	No reason identified
Territory 12	2/Low	9/High	No reason identified
Territory 8	5/Mod	8/High	No reason identified
Territory 4	4/Mod	0/Low	No Lab
Territory 11	6/Mod	9/High	External funding to build up the lab
Territory 13	6/Mod	0/Low	No Lab
*Territory*	*S2*	*S26*	
Territory 18	5/Mod	0/Low	No reason identified
Territory 12	2/Low	10/High	No reason identified
Territory 4	4/Mod	0/Low	Although some funds are available, there is no response capacity in case of an outbreak. Also no procedures established

Group 2	Statement pairs		Reason for inconsistency
*Territory*	*S1*	*S18*	
Territory 18	6/Mod	0/Low	No reason identified
Territory 4	6/Mod	0/Low	Although there is staff, there are no surveillance activities being performed
Territory 10	5/Mod	9/High	No reason identified

Group 3	Statement pairs		Reason for inconsistency
*Territory*	*S10*	*S19*	
Territory 5	4/Mod	1/Low	Although feral pigs are hunted regularly through a pig control program, there is no laboratory therefore samples are not collected

Territories identified as inconsistent in both methods for each pair of statements from the questionnaire that were identified for internal consistency analysis. Statements scored between 0 and 3 by respondents were ranked as Low Preparedness; statements scored between 4 and 7 were ranked as Moderate Preparedness; and statements scored between 8 and 10 were ranked as High Preparedness.

### Thematic analysis

Textual responses to the questionnaire were evaluated thematically to identify common gaps in prevention, detection, and outbreak response capabilities in the responding territories. Six themes were identified, with up to six sub-themes in each. The themes identified were communication and coordination; pig production systems; prevention and control; resources; surveillance; and traceability. Although these themes are all inter-related and often highlight similar gaps, the themes were selected to highlight frequently reported gaps identified through thematic analysis. See Additional Files 5 and 6 for the references and full thematic analysis. When a respondent mentioned an issue in multiple responses (i.e., insufficient staffing due to poor funding, inadequate surveillance due to poor funding), that respondent was counted once in the number of respondents reporting insufficient funding as a gap. Some respondents mentioned several issues in a single response, such as lack of funding and lack of training; in these instances, that comment was coded to all relevant themes. Some responses indicated success in one area but gaps in another, closely related, area, such as good communication with commercial swine producers and poor communication with backyard swine producers; these responses were coded to both relevant codes.

### Communication and coordination

Communication and coordination between governments, non-governmental organizations (NGO), intergovernmental organizations (IGO), and the private sector is important in disease prevention, detection, and response; gaps in these areas were highlighted by responses to the questionnaire. Three subthemes were identified in this theme: disease alerts and reports; government, IGO, and NGO communication and coordination; and government and private sector communication and coordination. Regarding disease alerts, some respondents reported having a system in place to report disease alerts or regular reports to the public (5 respondents). Some respondents noted that disease alerts or reports are generated internally on a regular basis, but are not publicly available or are only publicly available in certain circumstances, such as an active outbreak (6 respondents):

*“If there is any outbreak or concern, the Vet Services would produce monthly reports.”* (Territory 5)

Additionally, some respondents reported not having a specific or general communication plan (5 responses):

“*Currently, there is no communication plan in place, although there are future plans to establish one.*” (Territory 18)

*“No official reporting system or alerts available.”* (Territory 15)

Only 6 respondents mentioned alerts or communications specifically targeted to travelers. One respondent mentioned alerts being sent through social media and messaging applications, highlighting the importance of not only traditional media, but also social media sites in government communications. Thirteen respondents highlighted good coordination between IGOs, NGOs and the government, and between government agencies, while a lack of communication and coordination, or a need for improved communication and coordination, was mentioned by 11 respondents:

*“FAO, OIRSA and USAID provide financial support, training and monitoring to the swine sector.”* (Territory 6)

*“The participation of NGOs or non-governmental entities in direct support of pig farming is limited. More projects and aid have been seen for the bovine sector. Some international collaborations with Mexico have existed, but support for the pig sector is scarce.”* (Territory 10)

Eight respondents mentioned communication and coordination between the government and industry was good in at least some areas:

*“Although there were initial disagreements, education and awareness raising have improved the attitude of producers towards surveillance and prevention measures. Commercial producers are more receptive; however, the backyard sector presents greater difficulties, especially in indigenous communities in southern [territory] where pigs are raised free-range. Greater support from the [agriculture department] is required to improve practices.”* (Territory 10)

*“The private sector collaborates strongly with the public sector.”* (Territory 8)

Some respondents, however, highlighted gaps in communication and coordination between the government and industry, indicating a need for relationship building in this area, especially with backyard pig farmers (12 respondents):

*“Although clinical signs are understood, fear of reporting persists.”* (Territory 6)

*“Stakeholders are not involved in drafting resolutions.”* (Territory 2)

### Pig production systems

Pig production systems was identified as a theme encompassing six subthemes relevant to ASF prevention, surveillance, and response: disease-control measures and biosecurity; feral pigs; movement patterns; poorly characterized production systems; well-characterized production systems; and farmer training. Some respondents reported some adequate disease control and biosecurity measures (8 respondents), while some respondents highlighted gaps (13 respondents):

*“Regulations prohibit feeding raw feed to pigs. Commercial operations use formulated feed and trucks are not allowed on the premises. Producers pick up feed at the farm gate.”* (Territory 14)

*“There are biosecurity measures in place on commercial farms that can be improved. Approximately 60–70% of commercial operations maintain acceptable biosecurity practices. The organization and professionalization of the industry is increasing, although there is still room for improvement.”* (Territory 10)

Highlighted gaps included heterogeneous compliance, especially among backyard or free-range systems, and weak enforcement of regulations. Many responses reported no systematic characterization of the feral pig population (10 respondents), while a few cited populations limited to certain regions, or subject to control programs (5 responses). Six respondents reported no feral or wild populations. Responsibility for wild suid population characterization and control commonly falls to environment/wildlife agencies, with limited routine data exchange to animal health. Several responses noted minimal or no systematic monitoring and reliance on hunters/NGOs or environmental agencies for ad hoc information. Movement patterns were not well characterized (11 respondents) outside commercial channels; well characterized movement patterns were noted by 5 respondents:

*“Pig movements between farms are neither recorded nor studied. Information is available only at slaughterhouses regarding the origin of pigs arriving for slaughter.”* (Territory 18)

*“Studies have been conducted, the most recent in 2020, which allow us to understand movement patterns. Currently, there are new regulations that facilitate inspection and control of movement between districts, implemented for about a month now. There is a clear understanding of how animals are moved within the territory.”* (Territory 10)

Respondents described movement gaps as informal, with limited data capture and reliance on abattoir records. Persistent gaps were reported for backyard (12 respondents) and commercial (9 respondents) sectors in characterization of the industry, including varied categorization criteria. Respondents report a lack of current registries or censuses (in some cases the last census dates back to 2008). Eight respondents reported characterization of commercial pigs, and 6 reported characterization of backyard pig populations. Training of swine producers was reported as inadequate by 9 respondents, characterized by underreporting of suspect illness or mortality, fear of sanctions, and uncertainty about compensation:

*“Although there were initial disagreements, education and awareness raising have improved the attitude of producers towards surveillance and prevention measures. Commercial producers are more receptive; however, the backyard sector presents greater difficulties, especially in indigenous communities in southern [territory] where pigs are raised free-range. Greater support from the [government organization for agriculture] is required to improve practices.”* (Territory 10)

*“Although there is communication with the veterinary authority, some producers are reluctant to report suspected cases.”* (Territory 6)

Five respondents reported that producers were well trained to report suspicious clinical signs, with some reporting active engagement of extension services or industry groups to improve reporting:

*“The swine industry has a “Pork Consulate” and has participated in the [animal health association]. There are about 50 commercial producers out of a membership of approximately 6,000 in the livestock association, but many are not exclusively dedicated to swine farming. Joint workshops have been held with the government organization for agriculture] and producers, including with other countries (e.g. [territory]), to address ASF, CSF and other diseases.”* (Territory 10).

### Prevention and control

Disease prevention and control emerged as a theme with three subthemes: prevention plans to prevent the entry of a disease; contingency plans to respond to a disease incursion to stop it from spreading through the industry and wildlife; and regulations or legislative frameworks. Some territories reported having an updated prevention plan or prevention measures (3 respondents), but many highlighted gaps in prevention planning or implementation (13 respondents).

*“After the OIRSA AR, an AR was carried out in [territory], and based on the results, the Prevention Plan was adjusted.”* (Territory 11)

*“There is a national prevention plan that needs updating. There is a national focal point in transition, but there is no specific committee or working group beyond the [animal health authority]. The current plan is old (2020) and needs to be reviewed and modernized.”* (Territory 10)

Five respondents reported that a risk analysis had been recently completed, while others reported no recent risk analyses had been performed (8 respondents). Although one respondent reported an updated contingency plan, others reported the contingency plan needed to be improved or updated (11 respondents).

*“There is a plan focused primarily on prevention, passive surveillance and health education, but it lacks integration for a comprehensive response.”* (Territory 6)

Five territories noted the presence of legislation or regulations in place that promoted effective prevention or control measures, although some that reported adequate legislation in one area noted gaps in another area of veterinary legislation.

*“The legal framework is based on the Act that gave rise to [the animal health authority] in 2000, updated in 2020, and on secondary legal instruments (regulations and bylaws). [The animal health authority] has the authority to regulate and enforce regulations related to PPA and PPC, including the prohibition of feeding raw waste.”* (Territory 10)

*“Regulations include a ban on feeding raw waste and import restrictions, but no truck disinfection requirements. There are also quarantine and animal health certification protocols.”* (Territory 6)

Eleven territories reported a lack of comprehensive legislative framework, with some reporting gaps in existing legislation and others reporting a complete lack of legislation or a lack of enforcement.

*“Animal Health Act in place, but there are still political hurdles to pass before action.”* (Territory 5)

### Resources

Adequate resources are necessary for disease prevention, detection, and control; gaps in resources emerged as a theme with four subthemes: funding and supplies, laboratory capacity, staffing, and training. For funding and supplies, most respondents indicated insufficiency (13 respondents), pointing to structural shortfalls in which routine work may be sustained but surge capacity was fragile, whereas sufficiency was reported infrequently (3 respondents):

*“There are limited funds allocated to veterinary services, supplemented by NGO support. However, the lack of proactive actions makes these funds insufficient.”* (Territory 6)

*“Insufficient resources for primary prevention (entry points). Sufficient for surveillance”* (Territory 1)

*“There is a complete lack of supplies, resources, and personnel.”* (Territory 15)

*“In general, resources are available, but sometimes they are not used properly.”* (Territory 11)

Respondents frequently noted the absence of a dedicated funding source, reliance on emergency or external funds, and supply gaps for field operations. Laboratory capacity gaps ranged from fully absent to minimally adequate for initial detection (12 respondents), with recurrent constraints in equipment, space, and throughput. Even where PCR or ELISA were available, a single central laboratory was often a bottleneck for scale up, and in some cases there was reliance on laboratories in other countries for testing:

*“There is no laboratory in the country.”* (Territory 5)

*“The laboratories are underfunded. There is one central laboratory and three regional ones, but they are not fully operational.”* (Territory 6)

*“ELISA only, no PCR. Samples sent to [territory]”* (Territory 7)

Reports of fully equipped laboratories were reported by 4 respondents. Regarding staffing, responses emphasized understaffing (12 respondents), citing too few veterinarians relative to geography and workload, hiring freezes, and limited authority for private practitioners in emergencies:

*“The existing staff is trained, but insufficient. There are currently approximately 2 (4 in total in the agency) field veterinarians, with the estimated need for 8 to 10 veterinarians to cover the expanding pig population…”* (Territory 10)

*“There are few private veterinarians (about 2 consultants) working with the livestock sector. In the public sector, about 4 [animal health association] veterinarians cover the field… the lack of sufficient veterinary staff in the field can limit the ability to respond immediately.”* (Territory 10)

No responses indicated adequate staffing. Training was commonly described as uneven: public sector veterinarians and inspectors were consistently well-trained (9 respondents), while private veterinarians and laboratory personnel were more likely to be undertrained (5 respondents):

* “*More staff and more training are required…” (Territory 11)

*“[animal health association], which was born from outbreaks of CSF in [territory], has received extensive training.”* (Territory 8)

### Surveillance

Surveillance is an important component of disease detection, prevention, and control, providing information about disease presence and prevalence within a territory. Within the theme of surveillance, there were three different subthemes: differentials, general surveillance activities, and points of slaughter. Seven respondents stated there were no other differential diseases in the territory, with one respondent basing this response on the lack of massive mortality events rather than proven absence of disease:

*“Those diseases are not there, but this is an assumption based on no massive mortality events.”* (Territory 13)

Five respondents stated that there were differential diseases for ASF present in their territory. When respondents were asked to assess their territory’s surveillance activities, twelve respondents stated their territories had insufficient surveillance. Gaps were identified in surveillance of wild suid populations, identification and surveillance of high-risk populations, and passive surveillance:

*“Surveillance focuses on informal markets and border areas, identified as high-risk areas.”* (Territory 6)

*“Although they have agricultural protection services SEPA-OIRSA, their borders are very porous.”* (Territory 8)

*“Samples are rarely collected; passive surveillance is weak, and if something is suspected, samples must be sent to Plum Island for testing.”* (Territory 15)

Adequate surveillance was reported by five respondents in at least one capacity (for example, one respondent noted that passive surveillance is adequate, but targeted surveillance was lacking):

*“Surveillance plans are coordinated, including mandatory reporting, and agricultural organisations are actively involved in the response.”* (Territory 6)

*“Passive surveillance is being carried out. Risk-based surveillance is lacking.”* (Territory 8)

Only one respondent stated that no formal slaughter occurs. Nine respondents reported some formal slaughter occurring in their territories, however responses highlighted that informal slaughter also occurs, with a few commenting that even with formal slaughter as an option, many still choose to use informal slaughter (6 respondents):

*“No formal slaughter; no inspections conducted.”* (Territory 15)

*“There are formal slaughterhouses with trained inspectors, but many informal slaughterhouses still operate. FAO has supported improved reporting and access to information.”* (Territory 6)

*“Informal markets and clandestine slaughterhouses are recurring challenges.”* (Territory 6)

### Traceability

Eight territories had some form of traceability for live pigs, and three of those had some traceability for pork products; all three had systems that predominantly or solely focused on slaughter facilities, although one also had a system for traceability of imported semen:

*“There is traceability from the abattoir. There is no tracing for other movements that do not involve the slaughterhouse.”* (Territory 4)

*“Animals that enter the butcher station are entered in a database. At the moment it is only for goats and pigs. This is linked to the butcher facility.”* (Territory 17)

Three territories specifically noted a lack of either live pig (2 respondents) or pork product (1 respondent) traceability, and five territories noted a lack of effective traceability system for both live pig and pork product traceability:

*“There is traceability of live pigs, not of products.”* (Territory 2)

*“There is no tracing for other movements that do not involve the slaughterhouse.”* (Territory 4)

One respondent reported a traceability system that was provided by OIRSA, but it was outdated with limited use, although progress for between-farm traceability was also noted:

*“There is a traceability system provided by OIRSA, but it is outdated and has limited use. Progress has been made in tracking movements between farms.”* (Territory 6)

### Proposed capacity building mitigation strategies

Territories were given broad mitigation strategy categories of (1) training, (2) equipment, supplies, and logistical support, and (3) infrastructure, and were asked to generate strategies within those categories that would be beneficial if implemented in their territory; nine territories provided responses, four continental territories and five Caribbean territories. Responses are provided in Table 2. The mitigation strategies most commonly listed by territories included training and drills for veterinary staff and the need for improved infrastructure. Mitigation strategies that were least commonly listed as being of benefit were intergovernmental organization support of farmers and strengthened traceability capacity.

**Table 2:** **Mitigation strategies area**

	Percent (n)
*Training*	
Training and drills for veterinary and laboratory staff	89 (8)
Public education campaigns	22 (2)
Improved legislation and regulations	22 (2)
*Equipment, supplies and logistic support*	
Increased staff	22 (2)
Creating and maintaining a supply stockpile	22 (2)
Implementation of point of care tests	22 (2)
Increased funding	33 (3)
Increased operation or political support	22 (2)
Increased support of farmers by international institutions	11 (1)
*Infrastructure*	
Improved lab or veterinary services infrastructure	67 (6)
Acquiring and maintaining incinerators	22 (2)
Strengthened traceability capacity	11 (1)
Mobile or stationary quarantine facility	22 (2)

Capacity building mitigation strategies proposed by questionnaire respondents for prevention and control of ASF. Nine participants provided ideal mitigation strategies; the table lists the proportion and number (n) of those 9 participants listing mitigation strategies in each area.

### Site visit observations

Territories 15 and 18 site visits permitted validation of the questionnaire statement responses, with no inconsistencies identified between reported values and observations made during the site visit. (Additional File 7).

### Territory 14

The site visit permitted validation of the majority of the respondents’ ratings for the questionnaire (see Additional File 7). A discrepancy was noted in livestock management and communication, however. A high value was assigned for S5, “*The private pig industry is positive and receptive to the ASF control measures enacted by the Veterinary Authority,”* but it was noted on the site visit by the territory’s Veterinary Services personnel that producers show resistance to using biosecurity measures or updated farming practices which indicates that they are not receptive to ASF control measures. The questionnaire also had a high value listed for S25, “*A communication plan is in place and used to alert travelers about the risks of ASF introduction,”* meaning that the respondent was in agreement with this statement. But during the site visit it was noted that ASF signage was not present to alert travelers and passenger inspections for pork were inconsistent. The customs official also was unaware of ASF and the risk it poses in meat imports (see Additional File 7).

### Territory 16

The site visit validates most of the questionnaire responses, except for statements asking about pig production systems (see Additional File 7). A statement (S7) in the questionnaire read: “*Commercial pig producers are registered, and their location and pig stocks are known,”* which the respondent ranked as a 5. It was noted on the site visit that there are no commercial operations, only subsistence that are either confinement or extensive systems. While the locations of these farms are known, they are not commercial operations. A response of 0 was given to S6, “*Pig producers are classified into categories (e.g., highly technical commercial, small commercial, subsistence) based on defined criteria.”* While the territory’s Veterinary Services might not use a formally defined set of criteria, the producers are classified as defined above. The last discrepancy was for S21, “*High-risk populations are identified, and specific, risk-based surveillance is periodically carried out in these populations,”* which had a response of 0*.* It is known that the pig herds do practice garbage or swill feeding, classifying them as high-risk populations; however, no risk-based surveillance is conducted on them, indicating a low, but non-zero, score would be expected for this statement.

### Territory 17

There was one discrepancy between the site visit notes and the questionnaire. For S27 “*Biosecurity practices are common. The industry is organized and professional,”* the territory marked this statement with a 6, although on the site visit it was noted that there were no biosecurity measures in place when visiting farms. There was also no noted organization between the small number of farms with pigs.

## Discussion

The risk of ASF incursion to the Caribbean and Central and northern South America has been assessed previously [36, 45], but the preparedness of territories in the region to respond to an incursion of ASF has not been evaluated in a publicly available format.

The statistical analysis indicates distinct leverage points by region. Two of the continental participants showed consistently strong performance across legislative authority, producer engagement, laboratory readiness, and reporting systems, offering a practical benchmark for transferable routines such as clear legislative frameworks, routine passive surveillance, and timely public communication. Central and South American territories presented a generally mid-to-high profile with several leaders in preparedness with high self-reported scores; standardizing risk-based surveillance, tightening the linkage between surveillance design and laboratory throughput, and institutionalizing exercises with after-action learning are priority areas for capacity building. The Caribbean displayed the greatest dispersion, with many small jurisdictions reporting low agreement across multiple domains; the most actionable gaps were operational—tested contingency plans, stable reporting pipelines, basic movement and premises registries, and pre-arranged surge logistics (e.g., PPE, disinfection). The negative relationships between the absence of differential pig diseases (S24) and passive surveillance, disease-prevention regulations, risk-based surveillance, laboratory capacity, contingency plan testing, backyard pig population studied, and national ASF plan/governance should be interpreted cautiously, because an absence of ASF differential diagnoses may reflect limited surveillance or diagnostic capacity rather than true absence of these diseases. Collaboration with and between regional international organizations will continue to provide important capacity building and support for disease detection, prevention, control, and eradication [23, 25–29]. Additional support will be provided by the World Organization for Animal Health (WOAH) Global Framework for the Progressive Control of Transboundary Animal Diseases (GF-TADs), which published a new framework addressing ASF that intends to promote the capability of territories and regions to develop ASF prevention, control, and eradication programs customized to their regional situation and needs [46]. The GF-TADs framework emphasizes the need for strengthened Veterinary Services capacity, increased understanding and characterization of local pig production systems, enhanced collaboration and coordination between private, government, and nongovernmental organizations, education of farmers, and increased community engagement as some of the necessary components of a successful plan to address ASF [46]. Overall, a staged approach is warranted: operational readiness first in the Caribbean, consolidation and standardization in Central and South America, and anchor roles for the highest performing countries in protocols, mentorship, and surge diagnostics, performed within the GF-TADs framework created for global control of ASF.

Numerous gaps emerged in the thematic analysis in each of the six themes identified. A lack of adequate communication and coordination within governments, between governments and international partners, and between governments and stakeholders can hinder preparedness and response to emerging diseases such as ASF; building communication plans and facilitating relationship building between stakeholders, government agencies, and international partners would close this gap [47–50]. Communication and coordination between governments, IGOs, and NGOs on disease surveillance and response activities is especially important for robust disease prevention and control in smaller or less-resourced territories; such coordination allows territories to access expertise, funding, and resources they otherwise would not have access to [47]. Just as coordination between the government and intergovernmental and non-governmental organizations is important, adequate communication and coordination between the government and the private sector is essential to effective disease prevention and control, and providing alerts of disease outbreaks and surveillance activities helps keep the public informed, and helps fight misinformation [48, 50].

Gaps in pig production systems included poor disease control measures, inconsistent biosecurity across the pig sector, poor characterization of the industry, including movement patterns, inadequate farmer education around ASF, and the presence of feral pigs. By targeting improved characterization of the industry, governments can better understand where the highest risk areas for ASF incursion are, and they can more effectively prevent incursion and respond should an incursion occur [51, 52]. Building the biosecurity and movement control (quarantine, zoning, standstills, and permitting of movements) capacity of the pig sector would also help to address this gap, by reducing the risk that ASF incursion will occur [51, 53]. Outreach to farmers to discuss the risks of ASF and potential preventive measures is a potential measure to reduce the risk of incursion, especially if ASF enters the feral pig population [54–56]. Feral pigs pose a significant risk where they are present, as they can act as reservoirs for ASF and control of ASF spread once it enters a wild population is difficult [55, 57].

Veterinary legislation, risk analyses, prevention and contingency plans, and exercises to test the written plans are important aspects of preparedness [49, 58, 59]; gaps in ASF prevention and control that were highlighted in the thematic analysis centered around a lack of one or more of these aspects of preparedness. Prevention plans encompass activities that are undertaken to prevent entry of a pathogen [58]. Risk analyses are utilized before establishing or updating plans to identify territory specific higher-risk pathways of introduction and areas to focus on when preparing prevention and contingency plans [49]. After the incursion of ASF into a territory, contingency plans are integral to preventing further movement of the virus and achieving free status as quickly and efficiently as possible [49, 59]. Although risk analyses and writing and testing prevention and contingency plans are essential for addressing risk of disease incursion, veterinary specific legislation is also a concerning gap in some territories where it is lacking, as it is the tool that grants authority to the territory’s Veterinary Services to perform preventive and control measures adequately [58, 59]. International ASF response exercises present opportunities for multi-disciplinary collaboration and relationship building, and could be used to close gaps in prevention and contingency planning in territories where veterinary legislation is in place [58, 60].

The analysis also revealed gaps in funding and supplies, laboratory capacity, staffing, and training of lab personnel and private veterinarians. Without adequate resources, prevention and control activities will likely be implemented less effectively than they would be with adequate resources. An adequately trained and staffed veterinary services staff is essential to ensure an efficient and effective response to a disease outbreak, as they are both the boots on the ground and the decision makers in such a response [61–63]. Inappropriate or insufficient budget allocation before a disease outbreak occurs can hinder the response capacity, and additional budgetary inefficiencies have been shown to hinder response capacity [62–64]. Insufficient laboratory capacity, or a complete lack of laboratory capacity, hinders a territory’s ability to adequately diagnose and control an exotic animal disease in a timely manner; sending samples to a single lab that lacks surge capacity, or to a lab across international borders, may delay disease diagnosis, resulting in a delay in response [65]. Ensuring adequate resources are available for prevention of disease incursion, and for response in the event of an outbreak, is vital for the success of prevention and control of exotic animal diseases. In island territories, a lack of resources is further complicated by the logistics of acquiring supplies rapidly; supply imports may take weeks to be received, highlighting the importance of procuring adequate stockpiles and ensuring funding is in place to maintain it.

Gaps in surveillance activities were also identified. Some territories were aware of the presence of differentials for ASF in the territory, which could complicate diagnosis in the event of an outbreak and delay reporting if producers and veterinarians are not trained on recognizing ASF; other territories, however, assume no differentials are present due a lack of disease outbreaks, but do not perform active surveillance for differentials. Lack of surveillance of high-risk populations, at points of slaughter, and passive surveillance were also gaps that were identified by the thematic analysis. An important part of surveillance is monitoring for disease at certain points, with one of these being slaughter facilities; in informal slaughter, defined as slaughter done at locations other than regulated or monitored facilities, diseases can be missed [66, 67]. Surveillance activities are key to find and detect ASF when first introduced into a territory, and should include data capture, reporting, and analysis [52, 68]. All respondents providing textual responses reported gaps in traceability, highlighting the need for improved traceability systems and capacity building in this area. Increasing the capacity of these territories to perform surveillance and traceability activities would strengthen the territories’ ability to respond to and control outbreaks of numerous animal diseases, including ASF [69, 70].

Although gaps were highlighted in the discussion, many responses also highlighted strategies and areas where territories reported adequate preparedness; notably, no gaps in the training of Veterinary Services staff were noted. Additionally, many respondents noted that improvements were planned or being implemented to legislation, prevention and contingency plans, and surveillance.

Respondents identified mitigation strategies that would address the most pressing needs they noted in their territories, including training of staff, funding and supplies, political support or legislative power, international support, and increased infrastructure. Point of care testing, creation of a supply stockpile, and improved infrastructure were predominantly listed by Caribbean territories, but the remainder of the strategies were distributed more equally across the continental and Caribbean territories. Although training and infrastructure were most commonly reported as a mitigation strategy that would provide benefit, the variety of other strategies listed by one to three respondents highlights the unique needs of individual territories.

Site visits were performed to validate questionnaires, and to gain a deeper understanding of swine production in the Caribbean context. Of the islands visited, none had commercial pig stocks, although one is in the process of commercializing one of their pig farms. In two of the visited territories, observations made by researchers and the territories’ Veterinary Services personnel were in alignment with questionnaire responses; however, in three territories these observations did not align with one or more questionnaire responses. These observations centered around characterization of the pig industry and high risk populations, and the existence of communication plans targeting travelers. With a limited sample size for site visits, results cannot be extrapolated to the larger study population, but these discrepancies between questionnaire responses and site visit observations do suggest bias associated with self-reporting is likely to be present, which may falsely increase overall preparedness scores for individual territories; in some situations, it may also falsely decrease overall preparedness scores when a territory representative assigns a 0 score to a statement that site visit observations would suggest should be a low, non-zero score, such as having a baseline characterization of their high risk pig populations by the Veterinary Services knowing which pig farms are high risk and what their high risk practices are. As with all self-reported questionnaires, these biases should be kept in mind when evaluating the results and planning further research or interventions based on the results.

This research, although focused on preparedness of the included territories to respond to an incursion of ASF, can be applied more generally to other situations. The ability to effectively identify and trace outbreaks of disease, coordinate a disease response with industry, government, and international organizations, and prevent further outbreaks is applicable to other swine and livestock diseases. For example, similar preparedness capacities would be relevant for threats such as New World screwworm, avian influenza, and heartwater disease caused by Ehrlichia ruminantium, which are already present in parts of the Caribbean or the Americas and may pose risks of further spread among Caribbean territories, similar to the regional concern posed by ASF on Hispaniola [24]. In addition, an efficient response to animal health emergencies and other disasters relies on effective communication, coordination between multiple stakeholders, and the ability to provide resources. Increasing and improving these capacity building efforts do not need to be limited to target ASF preparedness, but should consider the overall preparedness of the territory to respond to pig diseases and non-disease disasters.

### Limitations

This study contains several limitations, which limit the strength of our findings. The questionnaires were self-assessments, and the responses may therefore be subject to extreme response bias or social desirability bias; some territories rated themselves highly for the numeric responses, and highlighted gaps in the textual responses that suggest they may have rated themselves too highly. Conversely, some smaller territories rated themselves quite low on the numeric responses; although these territories generally did have lower capacity, there is a possibility they rated themselves lower than warranted due to comparison with larger and better-resourced neighbors. In an effort to address this, an internal consistency evaluation was performed, but if a territory consistently rated themselves in a biased manner this would not eliminate the bias. Additionally, statements were open-ended and non-specific, which limited the strength of our analysis; as the textual responses were not required, the thematic analysis was also limited in scope. Eighteen out of forty-four invited territories responded to the survey, which may have resulted in non-response bias, where the non-respondents would have different opinions or outcomes than those that responded; our sample included a range of responding territories, including very small island nations, both sovereign and overseas dependencies, and large independent countries, which reduces the effect of this type of bias but does not eliminate it. No French territories were interviewed, presenting another potential source of non-response bias; this lack may have been due to language barriers or other causes. Although our sampling strategy utilized convenience sampling, because only those territories willing to participate in the study can be assessed, one may argue that the associated bias might be reduced, at least in part, by the large sampling fraction (18/44, 41% of the study population). The sample size for both mitigation strategies and site visits was small, and the convenience sampling strategy may have introduced bias in these components of the study.

## Conclusions

In conclusion, this analysis shows gaps in ASF preparedness across the Caribbean basin in resources, coordination, and operativity. Comparing responses, the continental territories had higher preparedness than Caribbean island territories, suggesting they may be positioned to act as regional supports to island territories; capacity building efforts should focus on building operational readiness in island territories through logistical support, private sector characterization, disease reporting systems, and simulation exercises to test prevention and contingency plans. Future efforts to evaluate the preparedness capacity of the region should focus on reaching territories that were not included in the present study and performing follow-up analysis to assess the efficacy of capacity building in the included territories.

## Supplementary Information

**Additional file 1. Questionnaire.** The preparedness questionnaire used in the survey presented here. Questions were phrased as statements and respondents were asked to rate their agreement with the statements on a scale of 1 to 10.

**Additional file 2. Statements Grouped for Internal Consistency.** Statements with answers that would be expected to be similar were identified for internal consistency analysis.

**Additional file 3. Numerical Questionnaire Responses.** The degree to which the territory’s representative agrees with the statement in Additional File 1, on a scale of 0–10, with 0 meaning no agreement and 10 meaning complete agreement. N/A means the statement is not applicable to the territory. CT means the territory is a continental territory; Cari means the territory is in the Caribbean.

**Additional file 4. Spearman Correlation of ASF Preparedness.** A heat map of pairwise Spearman rank correlations among the 30 ASF preparedness statements (S1–S30) scored 0–10 across 18 territories. Values closer to 1 indicate correlation, values closer to − 1 indicate divergence. 0 indicates no relationship.

**Additional file 5. Questionnaire Short Text Responses.** The text responses provided by the territories to each question in the questionnaire. Identifying information has been removed.

**Additional file 6. Thematic Analysis.** The full thematic analysis used in this work. Some responses were coded to multiple codes due to covering multiple topics or presenting areas of good preparedness and gaps in preparedness in the same response. Some territories had more than one response pertaining to each code, but in such cases the territory was only counted once.

**Additional file 7. Site Visit Validity.** Site visit observations to Territories 14, 15, 16, 17 and 18 for validation of responding territories’ questionnaire responses.

## Data Availability

All data generated or analysed during this study are included in this published article and its additional files.
