# Supplementary material for: Beneath the surface: a survey evaluating ASF preparedness across the Caribbean and Latin America
Source: Vet Res. 2026 Nov 13;57:203. doi: 10.1186/s13567-026-01886-9 (PMC13769168; doi:10.1186/s13567-026-01886-9)
Supplement: Supplementary file 1 [file 13567_2026_1886_MOESM7_ESM.pdf]

Additional File 7: Title: Site Visit Validity. Site visit observations to Territories 14, 15, 16, 17 and 18 for validation of respondents questionnaire responses.			
**Questionnaire Statement**	**Respondents Questionnaire Value (0 = strongly disagree with statement 10 = strongly agree with statement)**	**Site Visit Observations Agreement or Disagreement with Questionnaire Response**	**Site Visit Supporting Findings**
Territory 14			
High-risk populations are identified, and specific, risk-based surveillance is periodically carried out in these populations.	0	Agreement	Due to natural disasters there have been an emergence of feral livestock populations, composed of animals that were either released or abandoned. Only a small percentage are swine.
There is a legislative framework that grants sufficient authority to the veterinary authority to enact the required measures and provide adequate support.	6	Agreement	There is existing animal health legislation, but it is broad and not tailored to specific industries such as pig farming or pork trade. There is no formal pork export system, and current laws primarily aim to prevent free-roaming animals by requiring them to be tied or confined.<br>Current animal health regulations do not include detailed provisions for pork production or trade. Informal pork trade exists, notably the unofficial export of frozen pork to during holidays, which is not documented or regulated.<br>A visit to the local market showed a mix of pork products, primarily imported from the United States alongside some local pork.
A communication plan exists to alert producers about ASF risks and detections.	0	Agreement	Although the laws exist, implementation is challenging due to resource limitations and limited producer engagement.
Surveillance activities regularly include the country's wild boar and feral pig populations.	1	Agreement	Although the laws exist, implementation is challenging due to resource limitations and limited producer engagement.
The backyard pig population (number and geographical distribution) has been studied and characterized.	1	Agreement	Swine production is small-scale and primarily consists of backyard systems. There are an estimated 10 farms with fewer than 200 pigs total, but exact numbers are unknown due to inconsistent farmer reporting.

Biosecurity practices are common. The industry is organized and professional.	1	Agreement	Most swine producers show resistance to adopting biosecurity measures or modern farming practices. While outreach and education efforts are ongoing, meaningful changes are expected to take time.
The private pig industry is positive and receptive to the ASF control measures enacted by the Veterinary Authority.	8	**Disagreement**	Most swine producers show resistance to adopting biosecurity measures or modern farming practices. While outreach and education efforts are ongoing, meaningful changes are expected to take time.
Pigs are slaughtered in formal slaughterhouses with official inspection. Inspectors are well-qualified to identify and detect the disease.	8	Agreement	All local pigs are reportedly processed through a central slaughterhouse.
There are existing regulations aimed at preventing/controlling pig diseases, such as the prohibition of feeding pigs raw waste, the prohibition of bringing raw pork products into the country in luggage, mandatory washing/disinfection of live pig transport trucks, etc…inspection of pig products that come in.	1	Agreement	At the port of entry, passenger inspections for pork products are inconsistent. Signage at the airport primarily targets invasive species such as snails, insects, and snakes—no signage was present for African Swine Fever (ASF). One customs officer disclosed owning pigs that were not registered, and they were unaware of ASF altogether, reflecting significant training gaps among border personnel.
A communication plan is in place and used to alert travelers about the risks of ASF introduction.	9	**Disagreement**	At the port of entry, passenger inspections for pork products are inconsistent. Signage at the airport primarily targets invasive species such as snails, insects, and snakes—no signage was present for African Swine Fever (ASF). One customs officer disclosed owning pigs that were not registered, and they were unaware of ASF altogether, reflecting significant training gaps among border personnel.

There is sufficient staff at the national level (central, regional, and local) to carry out prevention and response activities for ASF, and the personnel are trained and qualified.	0	Agreement	At the port of entry, passenger inspections for pork products are inconsistent. Signage at the airport primarily targets invasive species such as snails, insects, and snakes—no signage was present for ASF. One customs officer disclosed owning pigs that were not registered, and they were unaware of ASF altogether, reflecting significant training gaps among border personnel.
Territory 15			
There is sufficient staff at the national level (central, regional, and local) to carry out prevention and response activities for ASF, and the personnel are trained and qualified.	1	Agreement	This territory has struggled to establish a formal veterinary governance structure resulting in significant gaps in veterinary oversight, legislation, and disease surveillance capacity.
Pigs are slaughtered in formal slaughterhouses with official inspection. Inspectors are well-qualified to identify and detect the disease.	0	Agreement	Very small pig population. These pigs are generally raised by individuals for subsistence or to share among friends and neighbors. The farms operate outside of any inspection or regulatory system, and there is no organized pork market or processing system.
Commercial pig producers are registered, and their location and pig stocks are known.	0	Agreement	Very small pig population. These pigs are generally raised by individuals for subsistence or to share among friends and neighbors. The farms operate outside of any inspection or regulatory system, and there is no organized pork market or processing system.
Biosecurity practices are common. The industry is organized and professional.	0	Agreement	Very small pig population. These pigs are generally raised by individuals for subsistence or to share among friends and neighbors. The farms operate outside of any inspection or regulatory system, and there is no organized pork market or processing system.
Farms are mainly confinement operations, and free-range pig farming is not allowed. Backyard pigs are adequately contained with fencing.	0	Agreement	Very small pig population. These pigs are generally raised by individuals for subsistence or to share among friends and neighbors. The farms operate outside of any inspection or regulatory system, and there is no organized pork market or processing system.

There is a legislative framework that grants sufficient authority to the veterinary authority to enact the required measures and provide adequate support.	0	Agreement	Political instability has hindered regulatory progress and delayed any substantive action to develop animal health legislation or formalize the veterinary infrastructure. Implementation of new regulations and such proposals have not advanced.
There are existing regulations aimed at preventing/controlling pig diseases, such as the prohibition of feeding pigs raw waste, the prohibition of bringing raw pork products into the country in luggage, mandatory washing/disinfection of live pig transport trucks, etc.	1	Agreement	Currently, there are no consistent import checks for animal products at airports or seaports. During outbreaks of African Swine Fever (ASF) in neighboring countries like the Dominican Republic, the only response has been the use of informational signage at points of entry. Enforcement of customs regulations is inconsistent and often depends on the discretion of individual customs officers. For instance, pig semen imports may be allowed or denied based on who is on duty, highlighting the need for standardized procedures.<br>Political instability has hindered regulatory progress and delayed any substantive action to develop animal health legislation or formalize the veterinary infrastructure.
There is good communication and predefined coordination mechanisms for public-private partnerships and within the government (border control, etc.) to support detection and response.	0	Agreement	Currently, there are no consistent import checks for animal products at airports or seaports. During outbreaks of African Swine Fever (ASF) in neighboring countries like the Dominican Republic, the only response has been the use of informational signage at points of entry. Enforcement of customs regulations is inconsistent and often depends on the discretion of individual customs officers. For instance, pig semen imports may be allowed or denied based on who is on duty, highlighting the need for standardized procedures.<br>Political instability has hindered regulatory progress and delayed any substantive action to develop animal health legislation or formalize the veterinary infrastructure.
Non-governmental institutions or groups collaborate with the government to support the pig production sector.	1	Agreement	This territory is not integrated into regional or international animal health reporting systems.
Territory 16			

Appropriate passive surveillance is carried out, including the collection and sending of suitable samples. When responding, the number of reported suspicions, the samples collected, the availability of diagnostic material, trained personnel, and the possibility of performing differential diagnoses must be considered.	0	Agreement	The staff is working on developing relationships with farmers and creating a program to better serve farmers and promote animal agriculture. This program when implemented would require monthly visits to each farm to act as passive surveillance and promote improvements.
There is sufficient staff at the national level (central, regional, and local) to carry out prevention and response activities for ASF, and the personnel are trained and qualified.	2	Agreement	There is a low number of staff working with livestock although they are well trained.
The private pig industry is positive and receptive to the ASF control measures enacted by the Veterinary Authority.	5	Agreement	Farmers are open to discussion and implementation of measures.
Commercial pig producers are registered, and their location and pig stocks are known.	5	**Disagreement**	There is a low number of farms with ⅔ being confinement and ⅓ being extensive systems. These are considered subsistence farms and not commercial. So while the locations are known they are not registered or commercial farmers.
Pig producers are classified into categories (e.g., highly technical commercial, small commercial, subsistence) based on defined criteria.	0	**Disagreement**	With the low number of known pig herds all are classified as subsistence either confinement or extensive. Although a defined criteria is not available.
Farms are mainly confinement operations, and free-range pig farming is not allowed. Backyard pigs are adequately contained with fencing.	5	Agreement	⅔ of the known farms are confinement operations. There currently are no regulations prohibiting free-ranging pigs.

There are existing regulations aimed at preventing/controlling pig diseases, such as the prohibition of feeding pigs raw waste, the prohibition of bringing raw pork products into the country in luggage. And In the country, feeding pigs with waste is not practiced and/or there are specific regulations for food treatment. Farms using trucks to transport food adequately disinfect and wash the trucks before they enter the farm.	0	Agreement	There are no regulations prohibiting waste feeding and it is practiced by the producers. An extensive farm only feeds scraps obtained from other farms that are cooked before being given to their pigs. Another farmer feeds uncooked vegetable and fruit scraps from restaurants and resort scraps to his pigs.<br><br>Import permits are required for the importation of live animals, but not for the importation of animal products.
High-risk populations are identified, and specific, risk-based surveillance is periodically carried out in these populations.	0	**Disagreement**	Swill or garbage fed pigs are a high-risk population and herds who use this practice are identified. Risk base surveillance is not conducted on these pigs that are swill fed.
There is good communication and predefined coordination mechanisms for public-private partnerships and within the government (border control, etc.) to support detection and response.	2	Agreement	Import permits are required for the importation of live animals, but not for the importation of animal products. The lack of import permits results in importation of products of unknown provenance without veterinary or other regulatory oversight. Due to the lack of import permits and clear directives for customs officials, shipments that do not match the manifest (i.e., a manifest states a shipment contains no pork products, but pork products are present) from the Dominican Republic are common.<br><br>Hazardous waste is disposed of in a specific part of the landfill, where it is meant to be disposed of in pre-dug holes that are then filled in; however, when the research team visited the landfill, hazardous waste was piled on the ground due to a communication breakdown resulting in no hole being pre-dug for disposal.

Slaughtering of pigs in formal slaughterhouses with official inspection.	0	Agreement	Regulations around antemortem examinations exist, but the veterinary staff only performs an estimated 4-5 antemortem inspections per year - primarily on cattle and the occasional suckling pig. A formal slaughter plant operated by the government is under construction, but is not currently operational, and is currently intended for slaughtering chickens and small ruminants, not pigs. Most slaughtering is apparently done by farmers or by the end-users who purchase live animals from farmers; however, no official reporting exists.
Territory 17			
Farms are mainly confinement operations, and free-range pig farming is not allowed. Backyard pigs are adequately contained with fencing.	8	Agreement	Territory 17 has very few pig production units. The setup is classic subsistence farming, with basic infrastructure, no biosecurity measures and multi species holdings, with pigs sharing farms with cattle, goats, chicken, etc. Animals are confined but able to move around in confinement.
Biosecurity practices are common. The industry is organized and professional.	6	**Disagreement**	The setup is classic subsistence farming, with basic infrastructure, no biosecurity measures and multi species holdings, with pigs sharing farms with cattle, goats, chicken, etc.
There are existing regulations aimed at preventing/controlling pig diseases, such as the prohibition of feeding pigs raw waste, the prohibition of bringing raw pork products into the country in luggage, mandatory washing/disinfection of live pig transport trucks, etc.	0	Agreement	The regulatory environment is not clear, is not well known and there is no clarity on how to proceed on multiple regulatory issues. The focus of the island is on the control of invasive species. Import permits are provided officially by the Veterinary Officer. Nevertheless, it was noted and discussed that it is common to have live animal imports to the territory without notifying the Veterinary Officer, thus facing a grey area when animals, although needing official Veterinary Officer approval for import, are brought to the island anyway by passing this authority. This has been observed for bovines and cats.

Farmers and veterinarians (private and public) are well-trained to detect and respond to ASF and commonly report suspicious clinical signs/mortality to the Veterinary Authority for investigation.	7	Agreement	As there are such few numbers of animals, diseased animals are rare and outbreaks even more so. In case of a sick animal the Veterinary Officer is called for immediate investigation. Formal disease surveillance is non-existent. In case of any diseased animal, farmers tend to report, but there is no established system for recording, processing and analyzing surveillance data
There is good communication and predefined coordination mechanisms for public-private partnerships and within the government (border control, etc.) to support detection and response.	2	Agreement	Communication gaps are huge, the formal channels in veterinary medicine are not clear, nor are the contact points and communication procedures. There are huge challenges in that regard. Limited contact also exists with nearby territories.
Territory 18			
There are existing regulations aimed at preventing/controlling pig diseases, such as the prohibition of feeding pigs raw waste, the prohibition of bringing raw pork products into the country in luggage, mandatory washing/disinfection of live pig transport trucks, etc.	4	Agreement	Currently, there are no specific national regulations or legislation tailored to pig farming, pork production, or trade, though general animal health laws are in place. Efforts are underway to develop and implement regulations focused on improving ASF prevention through training and stakeholder engagement.<br><br>For pork imports, border control relies primarily on self-declaration, especially for air and sea travelers. There is increased scrutiny for vessels and flights arriving from the Dominican Republic, given the ASF outbreaks there. On scheduled flights from the Dominican Republic, luggage inspections are carried out routinely, and any findings are reported directly. Border staff are aware that pork products are banned and have received training to support

Pig producers are classified into categories (e.g., highly technical commercial, small commercial, subsistence) based on defined criteria.	6	Agreement	The exact number of swine farms is unknown, but it is estimated that 20–30 farms practice pig production. Of these, only 10–20% are commercial operations, while the vast majority are subsistence-level family farms. The total pig population is estimated to be fewer than 20,000, inclusive of both commercial and backyard settings.
The backyard pig population (number and geographical distribution) has been studied and characterized.	4	Agreement	The exact number of swine farms is unknown, but it is estimated that 20–30 farms practice pig production. Of these, only 10–20% are commercial operations, while the vast majority are subsistence-level family farms. The total pig population is estimated to be fewer than 20,000, inclusive of both commercial and backyard settings.
Farms are mainly confinement operations, and free-range pig farming is not allowed. Backyard pigs are adequately contained with fencing. Comments: Confinement practices are generally lacking. Backyard pigs frequently roam freely, indicating inadequate fencing and containment practices across farms.	3	Agreement	The exact number of swine farms is unknown, but it is estimated that 20–30 farms practice pig production. Of these, only 10–20% are commercial operations, while the vast majority are subsistence-level family farms. The total pig population is estimated to be fewer than 20,000, inclusive of both commercial and backyard settings.
There is sufficient staff at the national level (central, regional, and local) to carry out prevention and response activities for ASF, and the personnel are trained and qualified.	6	Agreement	The department is understaffed making nationwide coverage and surveillance a challenge, but they are well trained.
Pigs are slaughtered in formal slaughterhouses with official inspection. Inspectors are well-qualified to identify and detect the disease.	7	Agreement	At slaughterhouses, ante- and post-mortem inspections are conducted by trained technicians, who are capable of identifying diseases or abnormalities.

There is a national ASF prevention/control plan. There is a national focal point dedicated to ASF and a national committee or working group on the disease, made up of trained and qualified individuals to create and carry out ASF response measures.	5	Agreement	For pork imports, border control relies primarily on self-declaration, especially for air and sea travelers. There is increased scrutiny for vessels and flights arriving from the Dominican Republic, given the ASF outbreaks there. On scheduled flights from the Dominican Republic, luggage inspections are carried out routinely, and any findings are reported directly. Border staff are aware that pork products are banned and have received training to support compliance.<br><br>They have a basic ASF emergency response plan in place. Current efforts focus on prevention, with limited capacity for outbreak management. If ASF were detected, the primary response strategy would involve culling infected animals. Concepts such as zoning and compartmentalization have been discussed but are not fully operationalized.
There is good communication and predefined coordination mechanisms for public-private partnerships and within the government (border control, etc.) to support detection and response.	4	Agreement	For pork imports, border control relies primarily on self-declaration, especially for air and sea travelers. There is increased scrutiny for vessels and flights arriving from the Dominican Republic, given the ASF outbreaks there. On scheduled flights from the Dominican Republic, luggage inspections are carried out routinely, and any findings are reported directly. Border staff are aware that pork products are banned and have received training to support compliance.
A contingency plan exists to respond to an ASF emergency, which has been tested in at least one simulation exercise.	0	Agreement	They have a basic ASF emergency response plan in place. Current efforts focus on prevention, with limited capacity for outbreak management. If ASF were detected, the primary response strategy would involve culling infected animals. Concepts such as zoning and compartmentalization have been discussed but are not fully operationalized.
